# A novel bacteriophage Tail-Associated Muralytic Enzyme (TAME) from Phage K and its development into a potent antistaphylococcal protein

**DOI:** 10.1186/1471-2180-11-226

**Published:** 2011-10-11

**Authors:** Vivek Daniel Paul, Sanjeev Saravanan Rajagopalan, Sudarson Sundarrajan, Shilpa E George, Jiya Y Asrani, Renjith Pillai, Ravisha Chikkamadaiah, Murali Durgaiah, Bharathi Sriram, Sriram Padmanabhan

**Affiliations:** 1 Gangagen Biotechnologies Pvt Ltd., No. 12, 5th Cross, Raghavendra Layout, Tumkur Road, Yeshwantpur, Bangalore 560 022, India; 2 Department of Molecular Genetics, University of Toronto,1 King's College Circle, Toronto, ON-M5S 1A8, Canada; 3 Lab Technologist, Pulmonary research, 559, Heritage Medical Research Center Dept of Medicine,112 St and 87 Ave, Edmonton, Alberta-T6G2S2, Canada; 4 Lupin Limited, Biotechnology R & D, Gat #1156, Ghotawade Village, Mulshi Taluka, Pune-411042, India

## Abstract

**Background:**

*Staphylococcus aureus *is a major cause of nosocomial and community-acquired infections. However, the rapid emergence of antibiotic resistance limits the choice of therapeutic options for treating infections caused by this organism. Muralytic enzymes from bacteriophages have recently gained attention for their potential as antibacterial agents against antibiotic-resistant gram-positive organisms. Phage K is a polyvalent virulent phage of the *Myoviridae *family that is active against many *Staphylococcus *species.

**Results:**

We identified a phage K gene, designated *orf*56, as encoding the phage tail-associated muralytic enzyme (TAME). The gene product (ORF56) contains a C-terminal domain corresponding to cysteine, histidine-dependent amidohydrolase/peptidase (CHAP), which demonstrated muralytic activity on a staphylococcal cell wall substrate and was lethal to *S. aureus *cells. We constructed N-terminal truncated forms of ORF56 and arrived at a 16-kDa protein (Lys16) that retained antistaphylococcal activity. We then generated a chimeric gene construct encoding Lys16 and a staphylococcal cell wall-binding SH3b domain. This chimeric protein (P128) showed potent antistaphylococcal activity on global clinical isolates of *S. aureus *including methicillin-resistant strains. In addition, P128 was effective in decolonizing rat nares of *S. aureus *USA300 in an experimental model.

**Conclusions:**

We identified a phage K gene that encodes a protein associated with the phage tail structure. The muralytic activity of the phage K TAME was localized to the C-terminal CHAP domain. This potent antistaphylococcal TAME was combined with an efficient *Staphylococcus*-specific cell-wall targeting domain SH3b, resulting in the chimeric protein P128. This protein shows bactericidal activity against globally prevalent antibiotic resistant clinical isolates of *S. aureus *and against the genus *Staphylococcus *in general. *In vivo*, P128 was efficacious against methicillin-resistant *S. aureus *in a rat nasal colonization model.

## Background

Peptidoglycan-degrading enzymes or murein hydrolases have the ability to digest bacterial cell walls. Such enzymes from bacteriophages represent a unique class of antibacterial agents because of their ability to cleave bacterial peptidoglycan in a species-specific or genus-specific manner. Thus, they provide a means to selectively target pathogens [1-3].

At the end of the bacteriophage infection process, progeny are released from the host cell by lysis, which is mediated by two phage-encoded gene products, endolysins and holins [4]. Holins are transmembrane proteins that create lesions in the cytoplasmic membrane through which peptidoglycan-degrading enzymes (endolysins) gain access to the peptidoglycan layer [4,5]. Bacteriophages encode another peptidoglycan-degrading enzyme involved in the initial stages of infection that facilitates phage DNA injection into the host cell. These proteins, which are distinct from endolysins, aid in the rapid lysis of host cells by a phenomenon referred to as "lysis from without" upon infection with high multiplicities of phage [6]. Enzymes involved in DNA injection are an integral component of the virion structure of many phages [7-9].

Examples of these phage structure-associated peptidoglycan-degrading enzymes include GP16 (phage T7), GP5 (phage T4), GP4 (*Salmonella *phage P22), GP3 (*Bacillus *phage Φ29), ORF50 (*Lactococcus lactis *bacteriophage Tuc2009), protein 17 (*Staphylococcus aureus *phage P68), and GP61 (*S. aureus *phage PhiMR11) [8-15].

*S. aureus *is an important human pathogen responsible for a wide variety of diseases and is a common cause of nosocomial and community-acquired infections. The emergence of antibiotic-resistant *S. aureus *strains underscores the need to develop alternate novel therapies [16-19]. In this context, we evaluated phage K, a known polyvalent phage with a broad host range that includes coagulase-positive and coagulase-negative staphylococci [20,21].

We report here the identification of the phage tail-associated muralytic enzyme (TAME) of phage K (PCT publication no. WO2007/130655: publication date November 15, 2007) [22] and generation of a chimeric protein that combines the lethal activity of TAME with the SH3b staphylococcal cell wall-binding domain of lysostaphin [23]. We demonstrated the efficacy of this chimeric protein *in vivo *using a rat nasal colonization model. Some of these findings were presented at the 2009 Madison Molecular Genetics of Bacteria and Bacteriophage meeting at the University of Wisconsin [24].

## Methods

### Bacterial strains, bacteriophages, plasmids, and growth conditions

All bacterial strains used in this study are listed in two tables (additional file 1, Table S1, additional file 2, Table S2). Cell culture media were obtained from HiMedia labs (India). Phage K was obtained from the National Collection of Type Culture (NC07814-02) and propagated on *S. aureus *RN4220 [25]. The methicillin-resistant *S. aureus *(MRSA) strain B911 was used for bactericidal activity assays, and RN4220 was used for zymograms.Plasmid pET21a (Novagen, USA) was used for cloning and the constructs were expressed under the control of a T7 promoter. Plasmid pRG5 (ATCC) carrying full-length lysostaphin was used as a template for amplifying the SH3b domain. All cultures were grown in Luria Bertani (LB) broth at 37°C, 200 rpm. Ampicillin (100 μg/ml) or isopropyl β-D-1-thiogalactopyranoside (IPTG, 1 mM) were added to the cultures as needed. All reagents used in this study were purchased from Sigma (USA) unless otherwise stated.

### Sequence analysis and Identification of TAME

The DNA sequence of phage K was obtained from the National Center for Biotechnology Information (NCBI) [GenBank: AY176327] [26]. Database searches were performed using BLASTN and BLASTP [27]http://www.ncbi.nlm.nih.gov. Domain identification and protein family allocation was performed with the Pfam database [28]http://pfam.sanger.ac.uk/ and the Conserved Domain Architecture Retrieval Tool [29]http://www.ncbi.nlm.nih.gov/Structure/lexington/lexington.cgi using the default parameters.

### Cloning of *orf*56 and its truncated forms

Phage K DNA was prepared as previously described [26]. All DNA manipulations were performed according to the methods of Sambrook and Russell [30]. Briefly, the full-length *orf*56 gene was amplified from phage K DNA by polymerase chain reaction (PCR) using a forward primer containing a unique NdeI site: 5'-CCGGAATTCCATATGCGTAGAATAAGACCTAAG-3' and a reverse primer incorporating an XhoI site: 5'-CCGCCGCTCGAGTTATTTCTTATCGTAAATGAATTGTGC-3'. Amplification was carried out using a Smart Cycler (BioRad, USA). The 2427-bp PCR product was gel-purified (GenElute™ gel extraction kit, Sigma, USA) and then digested with NdeI and XhoI. After gel purification, the DNA sequence was ligated into the pET21a vector. *Escherichia coli *DH5α cells were transformed with the ligation mixture, and transformants were selected on LB plates containing 100 μg/ml ampicillin. Plasmids (clones) were isolated from the transformants, screened by NdeI/XhoI digestion, and sequenced. The plasmid containing the full-length *orf*56 was designated as pGMB617. Truncated forms of *orf*56 were generated by PCR amplification using sets of primers for specific regions and cloned into the pET21a vector. Clone integrity was verified by restriction analysis and DNA sequencing.

### Construction of chimera P128

The DNA fragment encoding Lys16, excluding the stop codon, was PCR-amplified incorporating an NdeI site in the forward primer and XhoI site in the reverse primer. The fragment was cloned into the pET21a vector to generate pGDC108. The SH3b binding domain of lysostaphin was PCR-amplified from the plasmid pRG5 with XhoI restriction sites in both primers: forward primer 5'-CCGCCGCTCGAGACGCCGAATACAGGTTGGAAAACAAAC-3' and reverse primer 5'-CCGCCGCTCGAGTCACTTTATAGTTCCCCAAAGAAC-3'. The 300-bp PCR product was then cloned into pGDC108 to generate pGDC128. Transcription of the chimeric gene *Lys16-SH3b *in pGDC128 was driven by the T7 promoter.

### Protein expression and purification

The highly inducible T7 expression system of *E. coli *was used for hyperexpression of the target proteins. *E. coli *ER2566 (NEB Inc, MA, USA) harboring the different constructs was grown in LB at 37°C until absorbance at 600 nm (A_600_) reached 0.8, as determined by spectrophotometry (BioRad, CA, USA). Protein expression was induced by incubation with 1 mM IPTG at 37°C for 4 h. Cells were harvested by centrifugation at 7500 × *g *for 10 min, resuspended in 25 mM Tris-HCl (pH 7.5), and disrupted by ultrasonication. The cell lysate soluble and insoluble fractions were separated by centrifugation at 11000 × *g *for 15 min, and protein expression was analyzed by 12% polyacrylamide gel electrophoresis (PAGE). A crude soluble fraction containing the protein of interest was used for zymogram analysis and the bactericidal activity assay. After ammonium sulphate precipitation, soluble P128 was purified by two-step ion-exchange chromatography.

### Zymogram

Denaturing SDS-PAGE (Sodium Dodecyl Sulfate - Polyacrylamide Gel Electrophoresis) and zymograms were performed as previously described [31]. Briefly, muralytic activity was detected by separating protein samples by 12% SDS-PAGE on gels containing 0.2% of autoclaved *S. aureus *RN4220 cells. After electrophoresis, the zymograms were washed for 30 min with distilled water at room temperature, transferred to a buffer containing 25 mM Tris-HCl (pH 7.5) and 0.1% Triton X-100, and incubated for 16 h at 37°C for *in situ *protein renaturation. The zymograms were rinsed with distilled water, stained with 0.1% methylene blue and 0.001% KOH for 2 h at room temperature, and then destained with distilled water. Peptidoglycan hydrolase activity was detected as a clear zone against the dark blue background of methylene blue.

### Electron microscopy

Phage K particles were purified by CsCl density-gradient ultracentrifugation. Immunoelectron microscopy was performed by incubating approximately 5 × 10^8 ^phage particles with Lys16 antibodies conjugated to 10-nm gold particles (1:100) at room temperature overnight. The 1-ml samples were briefly centrifuged at 16000 × *g*, and the supernatant was collected and centrifuged at 16000 × *g *for 150 min. The resulting pellet was resuspended in 25 mM Tris-HCl (pH 7.5). A 20-μl aliquot of this sample was loaded onto Formvar-coated grids (TAAB Laboratories Equipment Ltd, UK) and dried. The grids were stained with 1% phosphotungstic acid and observed by transmission electron microscopy (Tecnai G^2 ^Spirit).

### Bactericidal activity assay

Bactericidal activity was assessed by measuring reduction in viable cells (CFU) after addition of P128 protein. The method is a modified version of the National Committee on Clinical Laboratory Standards assay used for determination of Minimum Bactericidal concentration [32]. Briefly, the MRSA clinical isolate B911 was grown in LB broth until A_600 _reached 1.0, and then an aliquot was diluted in LB broth to obtain 1 × 10^8 ^cells/ml. Aliquots (100 μl) were transferred to 1.5-ml microfuge tubes, treated with 100 μl crude or purified protein, and incubated at 37°C for 60 min at 200 rpm. Unless otherwise indicated, bactericidal activity was always performed using 10 μg/ml of P128. Residual viable cells were enumerated as colony-forming units (CFUs) by serial dilution and plating on LB agar plates.

### Turbidity reduction assay

Exponentially growing cells were harvested and resuspended in 25 mM Tris-HCl (pH 7.5). For gram-negative cultures, cells were pelleted, resuspended in CHCl_3_-saturated 50 mM Tris-HCl (pH 7.5), incubated for 45 min to expose the peptidoglycan layer, and then centrifuged at 3000 × *g*. The resulting pellet was resuspended in 25 mM Tris-HCl (pH 7.5), and the concentration was adjusted to about A_600 _of 0.8 for use as substrate for the assay. Purified P128 (50 μg/ml) was added, and A_600 _was determined at different time points (total assay volume 1 ml).

### *In vivo *efficacy of P128 in a rat nasal colonization model

Animal experiments were approved by the Institutional Animal Ethics Committee and the Committee for the Purpose of Control and Supervision of Experiments on Animals (CPCSEA). Gangagen is registered with CPCSEA (registration No. 1193/c/08/CPCSEA dated 21/4/2008). Healthy female Wistar rats (6-7 weeks old) were used in all experiments.

### Evaluation of commensal nasal flora

The commensal nasal flora of the rats was evaluated by nasal swabbing. Rat nares were swabbed by gentle insertion and withdrawal of a sterile Microbrush×(Microbrush^® ^International), which was moistened with sterile 0.85% NaCl. The swab portions of the Microbrush were cut and then completely immersed in 0.85% NaCl (150 μl) in microfuge tubes. Tubes were thoroughly vortexed, and the supernatant was diluted as needed and plated on agar containing 5% sheep blood. *Staphylococcus *colonies were identified based on morphology, biochemical tests and also analyzed using the HiStaph™ Identification kit (HiMedia). An *S. aureus*-specific enzyme-linked immunosorbent assay (ELISA) was used for confirmation.

### Experimental colonization of rat nares and evaluation of P128 efficacy

MRSA USA300 was grown overnight on nutrient agar containing 5% sheep blood. Colonies were harvested by flooding the plate with sterile 0.85% NaCl. Cells were pelleted by centrifugation (5800 × *g*, 10 min) and resuspended in sterile 0.85% NaCl (2 × 10^8^-5 × 10^8 ^cells/μl) for nasal instillation.

Rats were grouped and anaesthetized by intraperitoneal injection of ketamine (90 mg/kg body weight) and xylazine (9 mg/kg body weight). A 10-μl aliquot of *S. aureus *cell suspension was instilled into the nares of all animals on day 1. After 24 h, twice daily intranasal treatments to anaesthetized rats were initiated according to treatment group: P128 formulated as a hydrogel (50 mg/dose containing 100 μg P128), placebo gel that contained phosphate buffered saline in place of the protein, or Bactroban Nasal (30 mg/dose, 2% mupirocin ointment, GlaxoSmithKline). On day 3, the rats were euthanized by anesthetic overdose. The nasal tissue (except for the skin around the nares) was removed and processed for quantitative evaluation of colonization as described previously [33,34]. Aliquots of the supernatant (diluted as needed) were plated on nutrient agar containing 5% sheep blood and incubated overnight at 37°C. The *S. aureus *USA300 colonies were enumerated by tentative identification of hemolytic phenotype. Representative colonies from each USA300-positive animal were then purified on LB agar for biochemical characterization and confirmation by ELISA.

### Confirmation of *S. aureus *by ELISA

Purified colonies were suspended in 0.05 M carbonate-bicarbonate buffer (pH 9.6) to a cell density of about 1 × 10^9 ^cells/mL. A 200-μL aliquot of this cell suspension was used to coat 96-well plates and incubated overnight at 4°C. The wells were washed with Tris buffered saline with 0.1% Tween20 (TBST) and blocked with 1% bovine serum albumin (200 μL) in TBST for 1 h at 37°C. After repeated washes with TBST, rabbit polyclonal anti-RN4220 antiserum (100 μL, 1:20000) was added, and plates were incubated for 1 h at 37°C. The wells were washed again with TBST before adding alkaline phosphatase-labeled goat anti-rabbit antibody (100 μl, 1:5000). Plates were incubated for 1 h at 37°C. After washing the wells, the substrate *p*-nitro phenyl phosphate (100 μL) was added, the plates were incubated for 40 min, and absorbance at 405 nm was determined.

## Results

### Identification of TAME of phage K

**Figure 1:**
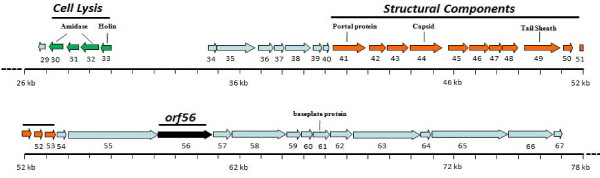
**Phage K genome**. A section of Phage K genome comprising the ORFs 29 to 67 is depicted. ORFs are indicated by colored arrows: putative lysis module (green), structural module (orange), proteins with a putative/hypothetical function (blue) and ORF56 (black).

Our bioinformatics analysis indicated that phageK harbors two genes involved in host cell wall lysis. In addition to the endolysin gene in the lysis cassette encoded by open reading frames (orfs) 30/32 [26], we found another gene (*orf*56) encoding a muralytic enzyme near the genes encoding structural tail proteins (Figure 1). *Orf*56 codes for a 91.2-kDa protein of 808 amino acids that possesses a C-terminal peptidoglycan-degrading domain (amino acids 678-808). We assigned this domain to the cysteine, histidine-dependent amidohydrolase/peptidase (CHAP) family through bioinformatic analysis (additional file 3, Figure S1) based on the reported characteristics of this domain [35].

BLASTP [27] searches revealed that ORF56 is related to the tail lysin protein ORF005 of *Staphylococcus *phage G1 and ORF007 of *Staphylococcus *phage Twort. A significant similarity was also found with GP98 of *Listeria *phage A511 (E value: 1e-120), GP29 of *Listeria *phage P100 (E value: 6e-120), putative tail lysin of *Enterococcus *phage PhiEF24C (E value: 3e-100), and putative tail lysin of *Lactobacillus *phage Lb338-1 (E value: 6e-53).

### Protein expression and activity of ORF56 and its N-terminal truncated forms

CHAP domain-containing proteins have been reported to be lytic to staphylococci [36]. Incubating 100 μl crude preparation of ORF56 with 1 × 10^7 ^cells of MRSA clinical isolate B911 for 60 min reduced CFUs by 90% compared with the control, demonstrating its bactericidal activity against *S. aureus *(additional file 4, Figure S2).

**Figure 2:**
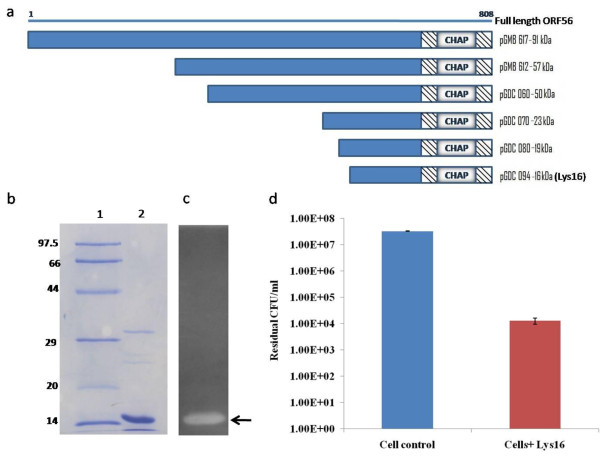
**ORF56 derivatives and purity profile, zymogram, and bactericidal activity of Lys16**. (a) Schematic representation of ORF56 and its N-terminal truncated forms. (b) SDS-PAGE profile of Lys16. Lane 1: molecular weight marker (97.5-14 kDa), Lane 2: purified Lys16 (5 μg). (c) Zymogram of purified Lys16 (5 μg) on autoclaved *S aureus *RN4220 cells. The muralytic activity of Lys16 is seen as a clear zone. (d) Bactericidal activity of Lys16. Purified Lys16 (100 μg/ml) reduced MRSA B911 viable CFUs by three orders of magnitude (99.9% cells killed).

To determine the function of ORF56, we cloned and expressed the full-length (2427-bp) *orf*56 gene. This yielded a 91-kDa protein as well as lower molecular-weight proteins, all of which showed muralytic activity on zymograms. This observation led us to generate truncated forms of ORF56 (57, 50, 23, 19, 16, and 13 kDa) (Figure 2a), all of which showed muralytic activity on zymograms and bactericidal activity against live *Staphylococcus *cells, except for the 13-kDa form, which was active only on zymograms (data not shown). The truncated 16-kDa ORF56, designated as Lys16 (Figure 2b), which showed cell wall-degrading activity on zymogram (Figure 2c) and lethal activity in *S. aureus *cultures (Figure 2d), was chosen for further characterization and development.

### Confirmation of ORF56 as TAME

**Figure 3:**
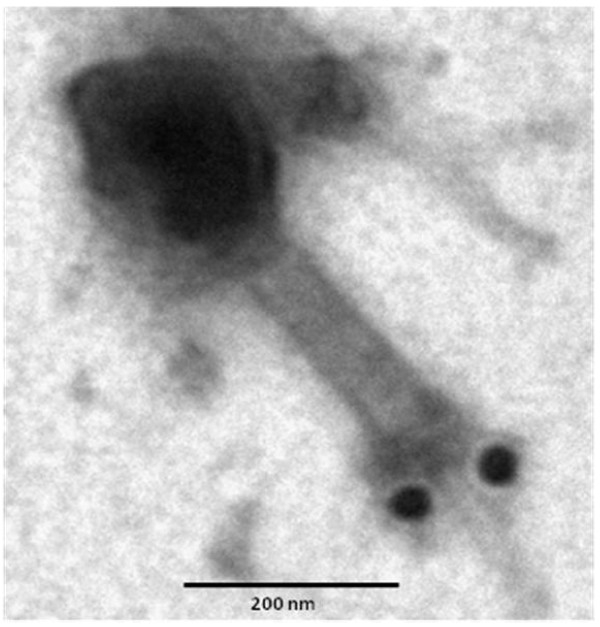
**Confirmation of ORF56-Lys16 as TAME of phage K by immunogold-electron microscopy**. Phage K particles were reacted with gold-conjugated polyclonal rabbit antibodies (10-nm immunogold particles) directed against Lys16 and subsequently negatively stained with phosphotungstic acid. Scale bar = 200 nm.

Immunogold electron microscopy was used to confirm that ORF56 is a part of the phage structure. We purified phage K by CsCl density gradient centrifugation and incubated phage particles with immunogold-labeled antibodies directed against Lys16. The gold-conjugated Lys16 antibody bound to the phage tail structure. This binding was confirmed to be specific (Figure 3).

### Antistaphylococcal chimeric protein P128

**Figure 4:**
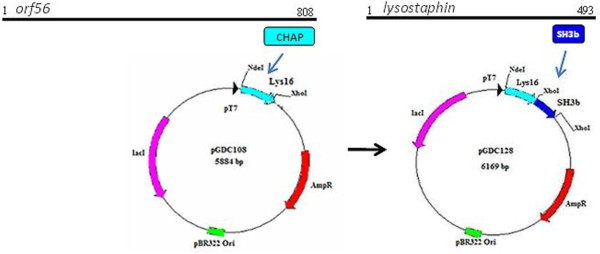
**Construction of chimera P128**. Schematic representation of the phage K *orf56 *gene showing the CHAP domain-encoding region and plasmid maps showing P128 construction. P128 was generated by fusing the Lys16 coding sequence that contains the muralytic CHAP domain of *orf56 *with the staphylococcal cell-wall targeting SH3b domain from lysostaphin.

**Figure 5:**
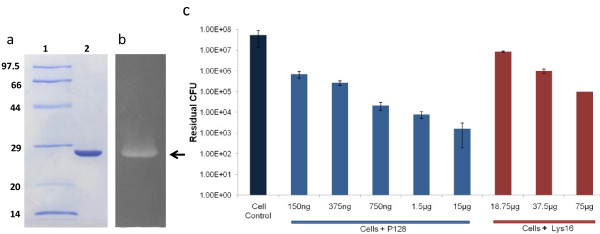
**SDS-PAGE profile and biological activity of P128 in zymogram and on live *S. aureus *cells**. (a) SDS-PAGE profile of P128. Lane 1: molecular weight marker (97.5-14 kDa), Lane 2: purified P128 (5 μg). (b) Zymogram of purified P128 (5 μg) on autoclaved *S. aureus *RN4220 cells. Muralytic activity of P128 is seen as a clear zone. (c) Varying concentrations of P128 was added to log-phase cells of MRSA B911 to evaluate biological activity on live cells. P128 was lethal at low (ng) concentrations. A 100-fold higher concentration of Lys16 was required for comparable activity.

We combined the muralytic protein Lys16 with SH3b [23], the staphylococcal cell wall-binding domain of lysostaphin, to generate the chimeric protein P128 (Figure 4). The cloned sequence was verified, and the chimeric construct yielded a protein of about 27 kDa. The soluble form of P128 was produced in *E. coli *and purified (> 95%). This protein showed muralytic activity on a zymogram with *S. aureus *cells (Figure 5a, b).

The bactericidal activity of Lys16 and P128 was compared by treating cells with varying concentrations of the protein and enumerating residual CFUs. P128 demonstrated superior antistaphylococcal activity compared with Lys16. At 750 ng/ml, P128 reduced viable cell numbers by three orders of magnitude. Lys16 did not achieve comparable activity, even at 100-fold higher concentration (Figure 5c).

### Specificity of P128 and dose-dependent activity

**Figure 6:**
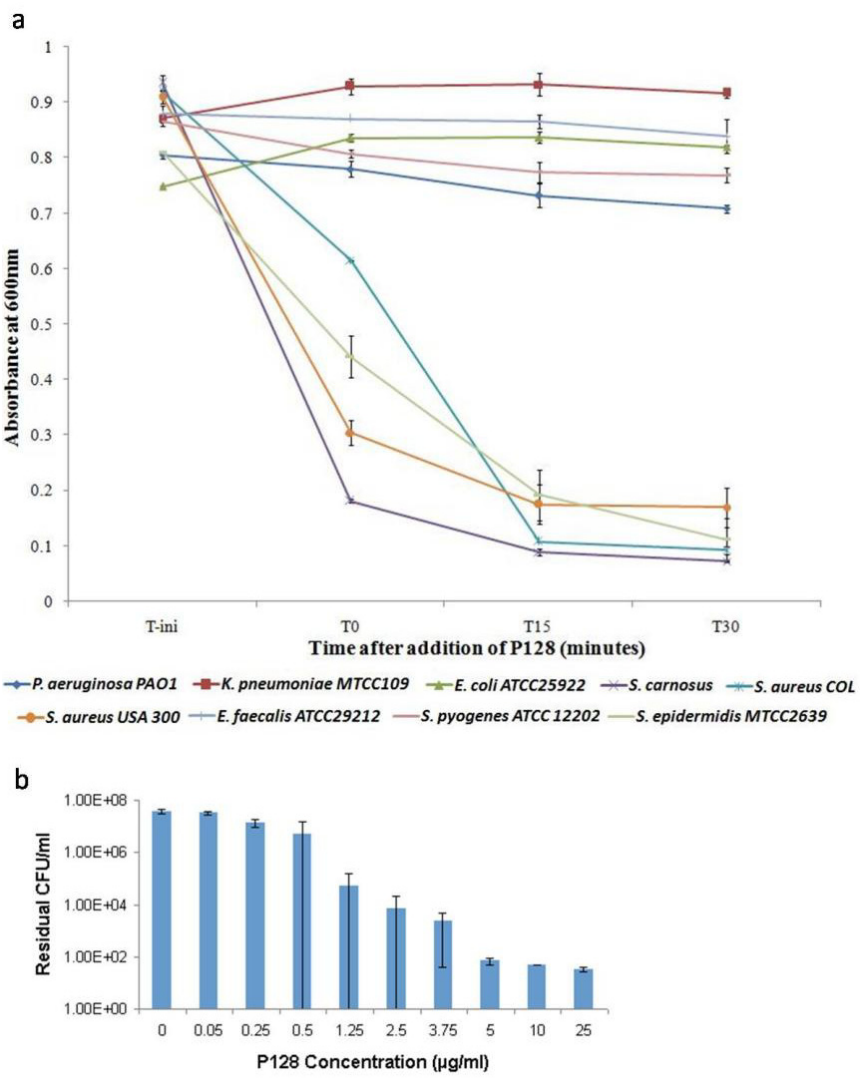
**Specificity and dose-dependent bactericidal activity of P128**. (a) P128 (50 μg/ml) was tested on log phase cells of gram positive and gram negative bacterial species by the turbidity reduction assay. P128 showed activity only against *Staphylococcus *species, which lysed rapidly after addition of the protein. No activity was observed against gram-negative bacteria or the other gram-positive bacteria tested. (b) The bactericidal effects of P128 are dose-dependent and 0.5 μg/ml was sufficient to reduce viable cell numbers by 90%. Reduction in viable cell numbers of over three orders of magnitude was observed in the concentration range of 2.5 - 25 μg/ml.

Purified P128 (50 μg/mL) was tested then for activity against *Escherichia coli*, *Enterococcus faecalis, Sterptococcus pyogenes, Staphylococcus epidermidis Klebsiella pneumoniae*, *Pseudomonas aeruginosa*, *Staphylococcus carnosus*, *Staphylococcus aureus COL*, and *Staphylococcus aureus USA300*. P128 was specific to Staphylococcus strains and caused significant reduction in the turbidity of the cultures, measured by optical density at 600 nm (A_600_). The cultures lysed within 15 minutes of addition of the protein. In contrast, the gram-negative cultures tested were not affected by P128 (Figure 6a).

The bactericidal activity of P128 against *Staphylococcus *strains was dose-dependent.

The minimum concentration of P128 required achieve > 99.9% killing was determined by a bactericidal activity assay with the MRSA COL strain. We found that concentrations ≥ 2.5 μg/mL killed > 99.9% of the cells (Figure 6b).

### Activity against global panel of *S. aureus *strains

**Figure 7:**
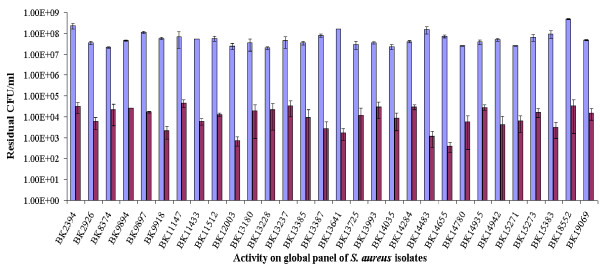
**Bactericidal activity of P128 on a panel of distinct clinical isolates**. Thirty globally represented *S aureus *clinical isolates consisting of MRSA and methicillin-sensitive *S. aureus *strains demonstrated sensitivity to P128 (10 μg/ml) with significant reduction in viable cell numbers. Blue bars represent cell controls and purple bars represent P128-treated cells.

To further characterize P128, its antimicrobial activity was tested on a panel of typed *S. aureus *strains, representing more than 3000 isolates worldwide. This panel included several MRSA strains and the clinically significant strains USA100, USA300, and USA400 (see additional file 1, Table S1). P128 reduced the cell numbers of these strains by 99% to 99.99%, demonstrating its efficacy against isolates of clinical significance (Figure 7).

### Experimental colonization of rat nares

Before initiating MRSA colonization, we evaluated the commensal bacterial flora of the rat nares in several experiments. In these Wistar rats, we found *Staphylococcus *strains including *S. sciuri subsp. rodentium*, *S. chromogenes*, and *S. equorum*; however, *S. aureus *was not detected (data not shown). Multiple experiments were performed using a total of 50 rats to establish the rate and the degree of colonization of MRSA USA300 on day 3 after instillation. Overall, 47 of 50 animals (94%) were colonized, and the median CFU recovered from the colonized animals was 2 × 10^4 ^(additional file 5, Table S3).

### Evaluation of P128 efficacy *in vivo*

**Figure 8:**
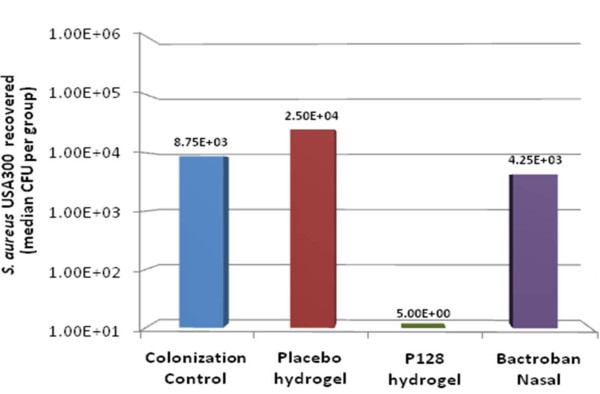
**Evaluation of P128 *in vivo *efficacy**. The median CFU number recovered in the P128 hydrogel-treated group was two orders of magnitude lower than that of the other groups.

At the time of treatment, one death had occurred in each of the placebo and P128 treatment groups. In the remaining rats, P128 hydrogel was found to completely decolonize four of nine (44.4%) animals (Table 1). Median CFU numbers recovered in the P128 hydrogel-treated group was two orders of magnitude lower than those of the other groups. All animals in the colonization control group and the groups treated with placebo or mupirocin remained colonized, as demonstrated by the large number of recoverable bacteria (Figure 8). To our knowledge, this is the first demonstration of efficacy against mupirocin-resistant community-associated MRSA USA300 in a nasal colonization model.

**Table 1:** MRSA colonization of rat nares after treatment

Group	Colonization (%)	CFUs recovered
**Colonization control**	10/10 (100)	2 × 10^3^-1.75 × 10^5^
**Placebo hydrogel**	8/9 (88.8)	1.5 × 10^2^-7.5 × 10^4^
**P128 hydrogel**	5/9 (55.5)	5 × 10^0^-7.5 × 10^3^
**Bactroban Nasal**	10/10 (100)	1.5 × 10^3^-2.53 × 10^4^

## Discussion

There has been considerable interest in phage endolysins as potential therapeutic targets. These cell wall-degrading enzymes play a role in releasing phage progeny at the end of the phage replication cycle. However, in this study we focused on enzymes capable of similar cell wall-degrading activity. These proteins are present as part of phage structure and are involved in the initial phase of phage infection. Phage tail-like bacteriocins produced by many *Pseudomonas *strains [37,38] kill other *Pseudomonas *strains by adsorbing to them and causing a fatal lesion in the cell envelope [39]. Both bacteriophages and phage-tail-like bacteriocins exert their lethal activity using a structural component. Structurally associated muralytic enzymes of phages have been identified at the base of the tail (e.g., T4 phage), within the phage head (e.g., T7 phage), in the internal membrane of the capsid (e.g., PRD1), or in the nucleocapsid (e.g., Phi6). The localization of the enzyme is associated with the distinct mode of cell entry used by each phage. Considering that TAMEs are part of the infection apparatus, they have a direct role in the specificity of phage-host interaction. These proteins are constantly exposed to environments encountered by the phage, suggesting that they are inherently stable. Phage TAMEs would therefore be generally well suited for antibacterial therapy. The focus of our study is such a structural protein, phage K TAME, which possesses bactericidal properties.

In this study, we identified a gene (*orf*56) within the structural module of the staphylococcal phage K genome that codes for a muralytic protein. We also carried out functional analysis of the gene product, which we designated as a TAME. The *orf*56 sequence is located in the tail gene cluster of the phage genome and shows significant sequence similarity with putative tail lysins of other phages of gram-positive bacteria. The catalytic region that confers bactericidal activity to ORF56 is localized to the C-terminal CHAP domain. We generated truncated versions of ORF56 by PCR- amplifying specific lengths of orf56 gene followed by cloning and expression. Zymogram analysis showed that the truncated forms of ORF56 were active against *S. aureus *peptidoglycan. Through this analysis, we identified the 16-kDa C-terminal region as the minimum portion of ORF56 required for bactericidal activity. This 16-kDa protein (Lys16) containing the CHAP domain was purified and found to be stable. Adding 100 μg/ml purified Lys16 to MRSA clinical isolates reduced cell numbers by 99.9%, demonstrating its antibacterial property (Figure 2).

Using antibodies against Lys 16, we were able to localize the protein on the phage tail structure. CHAP domains are present in a wide variety of proteins, including phage endolysins, bacterial autolysins, and various eukaryotic proteins. Most proteins that contain a CHAP domain function are peptidoglycan hydrolases and are associated with amidases [35,40]. No other known domains were identified in ORF56. Like the tail-associated lysin Tal2009, ORF56 undergoes autoproteolysis upon hyperexpression in an *E. coli *host [41].

Phage-encoded lytic enzymes typically have a modular organization consisting of a catalytic domain that degrades the peptidoglycan and a binding domain that recognizes the cell wall of the target bacterium [42]. However, no cell wall-binding domain could be identified in ORF56. NCBI BLAST [27] and Pfam [28] databases were used to compare cell wall targeting/binding domains of various *Staphylococcus *spp and their phages to select a suitable domain that could be fused to Lys16.

Our objective was to generate a chimeric protein with high specificity of target recognition and potent antistaphylococcal activity. To this end, we combined the muralytic activity of Lys16 with the known specific bacterial cell wall-binding SH3b domain from lysostaphin [23]. The chimeric protein P128 displayed higher activity than Lys16 and was found to be potent against *S. aureus *(Figure 4).

P128 was effective on a panel of MRSA and methicillin-sensitive *S. aureus *clinical isolates representing more than 3,000 isolates (Figure 7). In addition, we demonstrated the *in vivo *efficacy of P128 in a rat *S. aureus *nasal colonization model (Figure 8). We chose this model because growing evidence points to nasal carriage as the source of *S. aureus *infections in various clinical and community settings [43-45]. Although topical mupirocin is effective in clearing nasal *S. aureus *and reducing the incidence of infection, mupirocin resistance is limiting its preventative and therapeutic use [46,47]. In our study, we used USA300, which is a community-acquired mupirocin-resistant MRSA strain of high clinical significance [48]. To our knowledge, this is the first report of USA300 use in a nasal colonization model. P128 applied to rat nares in the form of an aqueous gel either decolonized the nares of USA300 completely or significantly reduced cell numbers. Thus, P128 is a novel chimeric protein with potent antistaphylococcal activity and warrants further development for therapeutic use.

## Conclusions

We report the development of a novel chimeric antistaphylococcal protein derived from phage K. The phage K gene, designated *orf*56 and encoding a TAME, was identified within the morphogenetic module of the phage genome. The 91-kDa gene product (ORF56) contained a sequence corresponding to the CHAP domain at the C-terminus. We cloned and expressed several N-terminal truncated forms of the *orf*56 gene to arrive at the smallest portion of the protein essential for antistaphylococcal activity. This 16-kDa protein (Lys16) was fused with an efficient staphylococcal cell-wall targeting domain (SH3b) derived from the bacterial protein lysostaphin to create the chimeric protein P128.

P128 shows specific activity against *Staphylococci *and lethal effects against *S. aureus *isolates of clinical significance and global representation. We tested the protein in an experimental nasal colonization model using MRSA USA300 and found it effective in decolonizing *S. aureus *in rat nares. Taken together, our findings show that P128 is a promising therapeutic protein candidate against antibiotic-resistant Staphylococci.

## Competing interests

Authors SP, VDP, SSR, and BS are inventors on the filed patent (Phage-derived antimicrobial agents: *International publication Number WO2007/130655*) describing methods and therapeutic compositions to reduce infections and methods for identifying additional such compositions. Authors have assigned rights to Gangagen Inc. which, is a current employer of BS, SS, SSR, SEG, RC, MD and a previous employer of SP, VDP, JYA, and RP.

## Authors' contributions

SP and BS participated in study design and coordination and contributed to data interpretation. VDP and SSR performed the sequence analysis, identification of TAME, and cloning and gene manipulation experiments. SP, SS, and SEG participated in clone construction. SEG, RC, and MD performed *in vivo *studies, and RP and JYA worked on the *in vitro *assays. VDP and SSR helped draft the manuscript. All authors read and approved the final manuscript.

## Supplementary Material

Additional file 1 **Table S1: Global panel of Clinical isolates received from The Public Health Research Institute Center (PHRI), New Jersey**. Click here for file

Additional file 2 **Table S2: Other strains used in the study**. Click here for file

Additional file 3 **Figure S1: Alignment of Phage K ORF56 with other CHAP domain proteins**. Click here for file

Additional file 4 **Figure S2: Bactericidal activity of ORF56**. Click here for file

Additional file 5 **Table S3: MRSA colonization status of rat nares 3 days after instillation of USA300**. Click here for file

## References

[B1] Schuch R Nelson D Fischetti VA A bacteriolytic agent that detects and kills Bacillus anthracis Nature 2002 418 884 889 10.1038/nature01026 12192412

[B2] Fischetti VA Bacteriophage lytic enzymes: novel anti-infectives Trends Microbiol 2005 13 491 496 10.1016/j.tim.2005.08.007 16125935

[B3] Loessner MJ Bacteriophage endolysins-current state of research and applications Curr Opin Microbiol 2005 8 480 487 10.1016/j.mib.2005.06.002 15979390

[B4] Young R Bacteriophage lysis: Mechanism and regulation Microbiol rev 1992 56 3 430 481 1406491 10.1128/mr.56.3.430-481.1992 PMC372879

[B5] Young R Bacteriophage holins: Deadly diversity J Mol Microbiol Biotechnol 2002 4 1 21 36 11763969

[B6] Delbruck M The growth of bacteriophage and lysis of the host J Gen Physiol 1940 23 5 643 660 10.1085/jgp.23.5.643 19873180 PMC2237944

[B7] Caldentey J Bamford DH The lytic enzyme of the Pseudomonas phage f6. Purification sand biochemical characterization Biochim Biophys Acta 1992 1159 44 50 10.1016/0167-4838(92)90073-M 1390911

[B8] Moak M Molineux IJ Role of the Gp16 lytic transglycosylase motif in bacteriophage T7 virions at the initiation of infection Mol Microbiol 2000 37 345 355 10.1046/j.1365-2958.2000.01995.x 10931329

[B9] Rydman PS Bamford DH Bacteriophage PRD1 DNA entry uses a viral membrane-associated transglycosylase activity Mol Microbiol 2000 37 356 363 10.1046/j.1365-2958.2000.01996.x 10931330

[B10] Kao SH McClain WH Roles of Bacteriophage T4 Gene 5 and Gene s Products in Cell Lysis J Virol 1980 34 1 104 107 6990015 10.1128/jvi.34.1.104-107.1980 PMC288675

[B11] Nakagawa H Arisaka F Ishii S Isolation and characterization of the bacteriophage T4 tail-associated lysozyme J Virol 1985 54 460 466 3157805 10.1128/jvi.54.2.460-466.1985 PMC254817

[B12] Moak M Molineux IJ Peptidoglycan hydrolytic activities associated with bacteriophage virions Mol Microbiol 2004 51 4 1169 1183 10.1046/j.1365-2958.2003.03894.x 14763988

[B13] Kenny JG McGrath S Fitzgerald GF van Sinderen DV Bacteriophage Tuc2009 encodes a tail-associated cell wall degrading activity J Bacteriol 2004 186 3480 3491 10.1128/JB.186.11.3480-3491.2004 15150235 PMC415775

[B14] Takac M Blasi U Phage P68 virion-associated protein 17 displays activity against clinical Isolates of Staphylococcus aureus Antimicrob Agents Chemother 2005 49 2934 2940 10.1128/AAC.49.7.2934-2940.2005 15980371 PMC1168661

[B15] Rashel M Uchiyama J Takemura I Hoshiba H Ujihara T Takatsuji H Honke K Matsuzaki S Tail-associated structural protein gp61 of Staphylococcus aureus phage φMR11 has bifunctional lytic activity FEMS Microbiol Lett 2008 284 1 9 16 10.1111/j.1574-6968.2008.01152.x 18462391

[B16] Smith TL Pearson ML Wilcox KR Cruz C Lancaster MV Robinson-Dunn B Tenover FC Zervos MJ Band JD White E Jarvis WR Emergence of vancomycin resistance in Staphylococcus aureus. Glycopeptide-intermediate Staphylococcus aureus working group N Engl J Med 1999 340 493 501 10.1056/NEJM199902183400701 10021469

[B17] Hiramatsu K Katayama Y Yuzawa H Ito T Molecular genetics of methicillin-resistant Staphylococcus aureus Int J Med Microbiol 2002 292 67 74 10.1078/1438-4221-00192 12195737

[B18] CDC Staphylococcus aureus resistant to vancomycin - United States MMWR 2002 51 565 567 12139181

[B19] Klevens RM Morrison MA Nadle J Petit S Gershman K Ray S Harrison LH Lynfield R Dumyati G Townes JM Craig AS Zell ER Fosheim GE McDougal LK Carey RB Fridkin SK Active Bacterial Core surveillance (ABCs) MRSA Investigators Invasive methicillin-resistant Staphylococcus aureus infections in the United States JAMA 2007 298 1763 71 10.1001/jama.298.15.1763 17940231

[B20] Rountree PM The serological differentiation of staphylococcal bacteriophages J Gen Microbiol 1949 3 2 164 73 18144968 10.1099/00221287-3-2-164

[B21] O'Flaherty S Ross RP Meaney W Fitzgerald GF Elbreki MF Coffey A Potential of the polyvalent anti-Staphylococcus bacteriophage K for control of antibiotic-resistant staphylococci from hospitals Appl Environ Microbiol 2005 71 1836 1842 10.1128/AEM.71.4.1836-1842.2005 15812009 PMC1082512

[B22] Padmanabhan P Paul VD Saravanan RS Sriram B Phage derived antimicrobial agents International publication Number WO2007/130655

[B23] Baba T Schneewind O Target cell specificity of a bacteriocin molecule: a C-terminal signal directs lysostaphin to the cell wall of Staphylococcus aureus EMBO J 1996 15 18 4789 97 8890152 PMC452215

[B24] Paul VD Saravanan S Asrani J Hebbur M Pillai R Sudarson S Sukumar H Sriram B Padmanabhan S A novel Bacteriophage Tail Associated Muralytic Enzyme (TAME) from PhageK and its development into a potent anti-staphylococcal chimeric protein In the Molecular Genetics of Bacteria and Phages Meeting, 4-9 August; Madison Wisconsin, USA

[B25] Kreiswirth BN Löfdahl S Betley MJ O'Reilly M Schlievert PM The toxic shock syndrome exotoxin structural gene is not detectably transmitted by a prophage Nature 1983 305 709 12 10.1038/305709a0 6226876

[B26] O'Flaherty S Coffey A Edwards R Meaney W Fitzgerald GF Ross RP Genome of staphylococcal phage K: a new lineage of Myoviridae infecting gram-positive bacteria with a low G+C content J Bacteriol 2004 186 2862 2871 10.1128/JB.186.9.2862-2871.2004 15090528 PMC387793

[B27] Altschul SF Madden TL Schäffer AA Zhang J Zhang Z Miller W Lipman DJ "Gapped BLAST and PSI-BLAST: a new generation of protein database search programs" Nucleic Acids Res 1997 25 3389 3402 10.1093/nar/25.17.3389 9254694 PMC146917

[B28] Finn RD Mistry J Schuster-Böckler B Griffiths-Jones S Hollich V Lassmann T Moxon S Marshall M Khanna A Durbin R Eddy SR Sonnhammer EL Bateman A Pfam: clans, web tools and services Nucleic Acids Research Database Issue 2006 34 D247 D51 10.1093/nar/gkj149 PMC1347511 16381856

[B29] Geer LY Domrachev M Lipman DJ Bryant SH CDART: protein homology by domain architecture Genome Res 2002 12 10 1619 23 10.1101/gr.278202 12368255 PMC187533

[B30] Sambrook J Russel DW Molecular Cloning: A Laboratory Manual 2001 Cold Spring Harbor Laboratory Press

[B31] Lepeuple AS Van Gemert E Chapot-Chartier MP Analysis of the bacteriolytic enzymes of the autolytic Lactococcus lactis subsp. cremoris strain AM2 by renaturing polyacrylamide gel electrophoresis: identification of a prophage-encoded enzyme Appl Environ Microbiol 1998 64 4142 4148 9797258 10.1128/aem.64.11.4142-4148.1998 PMC106620

[B32] National Committee for Clinical Laboratory Standards Methods for Determining Bactericidal Activity of Antimicrobial Agents; Approved Guideline 1999 Approved Guideline M26- A. NCCLS, Wayne, PA

[B33] Kiser KB Cantey-Kiser JM Lee JC Development and characterization of a Staphylococcus aureus nasal colonization model in mice Infect Immun 1999 67 5001 5006 10496870 10.1128/iai.67.10.5001-5006.1999 PMC96845

[B34] Kokai-Kun JF Walsh SM Chanturiya T Mond JJ Lysostaphin Cream Eradicates Staphylococcus aureus Nasal Colonization in a Cotton Rat Model Antimicrob Agents Chemother 2003 47 5 1589 97 10.1128/AAC.47.5.1589-1597.2003 12709327 PMC153340

[B35] Bateman A Rawlings ND The CHAP domain: a large family of amidases including GSP amidase and peptidoglycan hydrolases Trends Biochem Sci 2003 5 234 237 10.1016/S0968-0004(03)00061-6 12765834

[B36] Donovan DM Lardeo M Foster-Frey J Lysis of staphylococcal mastitis pathogens by bacteriophage phi11 endolysin FEMS Microbiol Lett 2006 265 1 133 9 10.1111/j.1574-6968.2006.00483.x 17054440

[B37] Kageyama M Shinomiya T Aihara Y Kobayashi M Characterization of a bacteriophage related to R-type pyocins J Virol 1979 32 951 957 117120 10.1128/jvi.32.3.951-957.1979 PMC525944

[B38] Nakayama K Takashima K Ishihara H Shinomiya T Kageyama M Kanaya S Ohnishi M Murata T Mori H Hayashi T The R-type pyocin of Pseudomonas aeruginosa is related to P2 phage, and the F-type is related to lambda phage Mol Microbiol 2000 38 2 213 31 10.1046/j.1365-2958.2000.02135.x 11069649

[B39] Shinomiya T Shiga S Bactericidal activity of the tail of Pseudomonas aeruginosa bacteriophage PS17 J Virol 1979 32 3 958 967 117121 10.1128/jvi.32.3.958-967.1979 PMC525945

[B40] Pritchard DG Dong S Barker JR Engler JA The bifunctional peptidoglycan lysin of Streptococcus agalactiae bacteriophage B30 Microbiology 2004 150 2079 2087 10.1099/mic.0.27063-0 15256551

[B41] Casjens S Hendrix R Calendar R Control mechanisms in dsDNA bacteriophage assembly: The Bacteriophages 1988 Kluwer Academic/Plenum Publishers 15 91

[B42] Loessner MJ Bacteriophage endolysins-current state of research and applications Curr Opin Microbiol 2005 8 4 480 7 10.1016/j.mib.2005.06.002 15979390

[B43] Kluytmans J van Belkum A Verbrugh H Nasal carriage of Staphylococcus aureus: epidemiology, underlying mechanisms, and associated risks Clin Microbiol Rev 1997 10 3 505 20 9227864 10.1128/cmr.10.3.505 PMC172932

[B44] von Eiff C Becker K Machka K Stammer H Peters G Nasal Carriage as a Source of *Staphylococcus Aureus *Bacteremia Study Group N Engl J Med 2001 344 11 6 10.1056/NEJM200101043440102 11136954

[B45] Lamers RP Stinnett JW Muthukrishnan G Parkinson CL Cole AM Evolutionary analyses of Staphylococcus aureus identify genetic relationships between nasal carriage and clinical isolates PLoS One 2011 21; 6 1 e16426 21283661 10.1371/journal.pone.0016426 PMC3025037

[B46] van Rijen M Bonten M Wenzel R Kluytmans J Mupirocin ointment for preventing Staphylococcus aureus infections in nasal carriers Cochrane Database Syst Rev 2008 8 4 CD006216 10.1002/14651858.CD006216.pub2 PMC8988859 18843708

[B47] Hogue JS Buttke P Braun LE Fairchok MP Mupirocin Resistance Related to Increasing Mupirocin Use in Clinical Isolates of Methicillin-Resistant Staphylococcus aureus in a Pediatric Population J Clin Microbiol 2010 48 7 2599 2600 10.1128/JCM.02118-09 20421433 PMC2897475

[B48] Han LL McDougal LK Gorwitz RJ Mayer KH Patel JB Sennott JM Fontana JL High Frequencies of Clindamycin and Tetracycline Resistance in Methicillin-Resistant *Staphylococcus aureus *Pulsed-Field Type USA300 Isolates Collected at a Boston Ambulatory Health Center J Clin Microbiol 2007 45 1350 2 10.1128/JCM.02274-06 17287335 PMC1865823

