# Supplementary material for: A novel bacteriophage Tail-Associated Muralytic Enzyme (TAME) from Phage K and its development into a potent antistaphylococcal protein
Source: BMC Microbiol. 2011 Oct 11;11:226. doi: 10.1186/1471-2180-11-226 (PMC3207973; doi:10.1186/1471-2180-11-226)
Supplement: Additional file 1 — Table S1: Global panel of Clinical isolates received from The Public Health Research Institute Center (PHRI), New Jersey. [file 1471-2180-11-226-S1.DOC]

**Additional File 1, Table S1: Global panel of Clinical isolates received from The Public Health Research Institute Center (PHRI), New Jersey.**

| ***S. aureus* strain #** | **Methicilin Resistance Element Type** | **Strain Description / Type** | ***spa* Type** | **Coagulase**  **status** |
| --- | --- | --- | --- | --- |
| BK2394 | - | 12 hospital study | 17 | - |
| BK2926 | - | USA100 and Japanese outbreak clone: N315 | 2 | - |
| BK8374 | - | COL, sequenced by TIGR | 1 | - |
| BK9894 | - | Boston, pediatric infection | 2 | - |
| BK9897 | MEC IV | USA 400, MW2, sequenced strain | 131 | POS |
| BK9918 | - | CA MRSA, Nebraska | 194 | - |
| BK11147 | Untyped | - | - | - |
| BK11433 | Untyped | - | - | - |
| BK11512 | - | MRSA252, sequenced English outbreak strain called EMRSA16 | 16 | - |
| BK12003 | Untyped |  | 19 | - |
| BK13180 | MEC IV | NJ CA-MRSA Soft Tissue Infections 2005 | 251 | NEG |
| BK13228 | MEC II |  | 1 | NEG |
| BK13237 | MEC II |  | 47 | NEG |
| BK13385 | MEC IV |  | 29 | NEG |
| BK13387 | MEC IV |  | 193 | NEG |
| BK13641 | MEC IV |  | 59 | POS |
| BK13725 | MEC IV |  | 7 | NEG |
| BK13993 | MEC II |  | 2 | NEG |
| BK14035 | MEC II |  | 2 | POS |
| BK14284 | MEC II |  | 33 | NEG |
| BK14483 | MEC II |  | 15 | NEG |
| BK14655 | MEC IV |  | 131 | POS |
| BK14780 | MEC IV |  | 17 | NEG |
| BK14935 | MEC IV |  | 1 | POS |
| BK14942 | MEC IV |  | 363 | POS |
| BK15271 | MEC IV |  | 2 | NEG |
| BK15273 | MEC IV |  | 35 | POS |
| BK15383 | MEC IV |  | 206 | POS |
| BK18552 | MEC IV | CA MRSA in Europe | - | - |
| BK19069 | MEC IV | USA300 sequenced strain | 1 | POS |
|  | | | | |

‘-’ : Data not available, NEG : negative, POS : positive
