# Supplementary material for: A novel bacteriophage Tail-Associated Muralytic Enzyme (TAME) from Phage K and its development into a potent antistaphylococcal protein
Source: BMC Microbiol. 2011 Oct 11;11:226. doi: 10.1186/1471-2180-11-226 (PMC3207973; doi:10.1186/1471-2180-11-226)
Supplement: Additional file 2 — Table S2: Other strains used in the study. [file 1471-2180-11-226-S2.DOC]

**Additional File 2, Table S2 : Other strains used in the study.**

| **Organism** | **Strain name** | **Source/Reference** |
| --- | --- | --- |
| *Staphylococcus aureus* | RN4220 (MSSA) | 1 |
| *Staphylococcus aureus* | B911(MRSA) | 2 |
| *Staphylococcus carnosus* | ATCC51365 | 3 |
| *Enterococcus faecalis* | ATCC29212 | 3 |
| *Escherichia coli* | ATCC25922 | 3 |
| *Streptococcus pyogenes* | ATCC12202 | 3 |
| *Klebsiella pneumoniae* | MTCC109 | 4 |
| *Pseudomonas aeruginosa* | PA01 | 5 |

^1^Dr. Richard Novick, Skirball Institute, New York , ^2^ This study, ^3^ATCC, ^4^MTCC , ^5^ Dr. Kalai Mathee, Florida International University, Miami, FL
