# Supplementary material for: A novel bacteriophage Tail-Associated Muralytic Enzyme (TAME) from Phage K and its development into a potent antistaphylococcal protein
Source: BMC Microbiol. 2011 Oct 11;11:226. doi: 10.1186/1471-2180-11-226 (PMC3207973; doi:10.1186/1471-2180-11-226)
Supplement: Additional file 3 — Figure S1: Alignment of Phage K ORF56 with other CHAP domain proteins. [file 1471-2180-11-226-S3.DOC]

**Additional File 3, Figure S1: Alignment of Phage K ORF56 with other CHAP domain proteins**


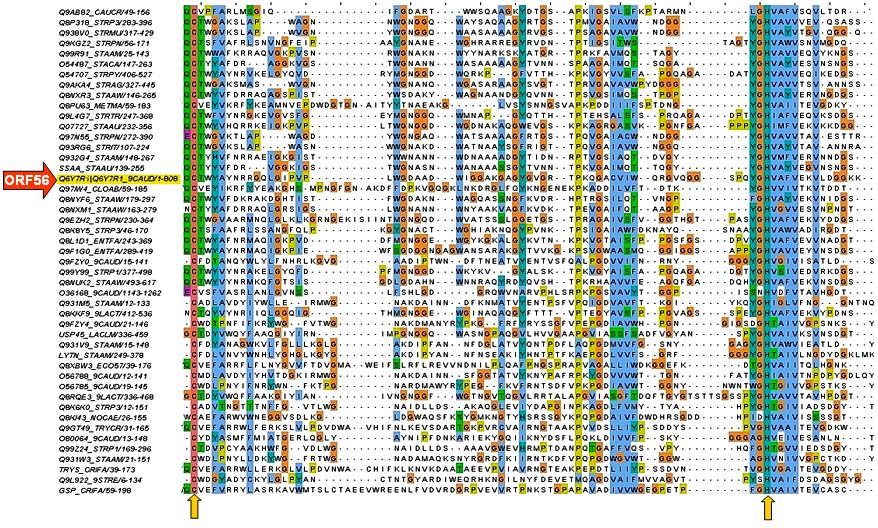


Figure S1 : Multiple sequence alignment of Phage K ORF56 with the conserved core sequence of other CHAP domain-containing proteins. CHAP domain contains two invariant residues, a Cysteine and a Histidine. These residues form part of the putative active site and are indicated. Protein identifiers are SWISS-PROT Accession numbers. Alignment was created with CLUSTALW using Pfam entry PF05257 containing seed alignment of 49 sequences.
