# Supplementary material for: A novel bacteriophage Tail-Associated Muralytic Enzyme (TAME) from Phage K and its development into a potent antistaphylococcal protein
Source: BMC Microbiol. 2011 Oct 11;11:226. doi: 10.1186/1471-2180-11-226 (PMC3207973; doi:10.1186/1471-2180-11-226)
Supplement: Additional file 4 — Figure S2: Bactericidal activity of ORF56. [file 1471-2180-11-226-S4.DOC]

**Additional File 4, Figure S2: Bactericidal activity of ORF56**

**
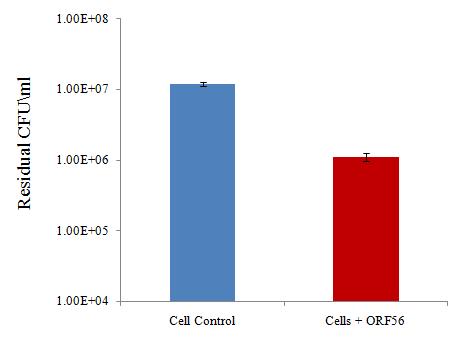
**

1 x 10^7^ cells of an MRSA strain (B911) were used to test activity. A crude preparation of ORF56 expressed in *E.coli* was added to B911 cells and incubated for 1 hour at 37ºC. P128-treatment resulted in ten-fold reduction of CFUs (90% cells killed) while untreated cell-control remained unchanged.
