# Supplementary material for: A novel bacteriophage Tail-Associated Muralytic Enzyme (TAME) from Phage K and its development into a potent antistaphylococcal protein
Source: BMC Microbiol. 2011 Oct 11;11:226. doi: 10.1186/1471-2180-11-226 (PMC3207973; doi:10.1186/1471-2180-11-226)
Supplement: Additional file 5 — Table S3: MRSA colonization status of rat nares 3 days after instillation of USA300. [file 1471-2180-11-226-S5.DOC]

**Additional File 5, Table S3: MRSA colonization status of rat nares 3 days after instillation of USA300.**

| **CFU (Range)** | **No. of Rats** |
| --- | --- |
| 0 to10 | 3 |
| 10 to 100 | 1 |
| 100 to 1000 | 3 |
| 1000 to 10000 | 15 |
| 10000 to 100000 | 21 |
| 100000 to 1000000 | 7 |
